# Indigenous Comprehension of Fodder Trees and Shrubs in Semiarid Areas of Metema District, Northwestern Ethiopia

**DOI:** 10.1155/tswj/6087166

**Published:** 2025-12-15

**Authors:** Yirgalem Melkamu, Getinet Masresha, Tiruye Ayenew, Asmamaw Alemu, Daniel Tadesse

**Affiliations:** ^1^ Department of Biology, University of Gondar, Gondar, Ethiopia, uog.edu.et; ^2^ Department of General Forestry, University of Gondar, Gondar, Ethiopia, uog.edu.et; ^3^ Department of Plant Science, University of Gondar, Gondar, Ethiopia, uog.edu.et

**Keywords:** fodder value, indigenous knowledge, Metema District, trees and shrubs

## Abstract

**Background:**

Trees and shrubs are a major component of the diet for livestock in Ethiopia, although they were not fully documented in many parts of the country. Thus, the study was undertaken to assess fodder trees and shrubs in the semiarid area of Metema District, northwestern Ethiopia.

**Methods:**

Eight Kebeles were selected purposively based on livestock production and vegetation availability. Cochran′s formula was used to select 385 informants composed of 40 purposively selected key informants and 345 systematically selected general informants. Data were collected through interviews, guided field walks, and focus group discussions. Then, they were analyzed using descriptive statistics, Jaccard′s coefficient of similarity (JCS), preference ranking, paired comparison, and relative frequency of citation. Knowledge difference between informant types and age groups was analyzed using *t*‐test and Pearson′s correlation, respectively.

**Results and Discussion:**

Livestock production is the main source of livelihood for Metema District communities. Goats were the most reared animals per household (34), followed by cattle (26). To feed the livestock, 46 fodder trees and shrubs within 36 genera and 24 families were identified. Fabaceae was the most species‐rich family (17.39%), followed by Combretaceae (13.04%). Key informants reported significantly more fodder species than general informants (*p* = 0.001). They also have higher information consistency on accessing fodders. A slightly positive correlation on the number of fodder species was also observed between age groups. Leaves were the dominant fodder plant parts. *Pterocarpus lucens* was the most cited, preferred, and valuable fodder species. Goats browsed 100% of the recorded fodder plants. Most fodder trees and shrubs (91.3%) were sourced from wild environments.

**Conclusions:**

This study revealed that the indigenous communities of Metema District possess high knowledge of tree and shrub fodder plants. The plants are vital components of their livestock feeding systems to improve their livelihoods.

## 1. Background

Indigenous knowledge reflects innovations, practices, and beliefs developed and adapted by a particular indigenous people through interaction and experience with the environment [1]. This includes the knowledge developed by a particular group through long‐term interaction with plants for their medicinal, food, fodder, and other values. A review of the literature demonstrates that there has been a growing interest in indigenous knowledge of pastoral systems [2–4]. Pastoral knowledge of fodder plants is common in developing countries like Ethiopia where most of the population experienced pastoral and agropastoral economic activities in its broad semiarid regions [4, 5]. In this area, trees and shrubs are the most diversified plant habits [6] used as a major component of the diet for livestock especially during dry seasons and fodder stress periods [7]. This has resulted from spatial and seasonal shortages of pasture, increasing livestock numbers and shortages of grazing lands. Various parts of shrubs and trees especially leaves, pods, seeds, and edible twigs are used as supplementary feeds for animals [8]. Browsing animals like goats and camels are almost totally dependent on trees and shrubs for their nutritive requirements. Cattle and sheep also complement their diet with these plants for different nutrients [9]. These livestock populations prefer fodder trees and shrubs differently depending on the livestock type and the different fodder properties of the plants [8, 10].

Among the Ethiopian semiarid environments, Metema District is the one characterized by having an agropastoral way of life. In this area, grassy and herbaceous species are available sometimes after the onset of rain and last for short periods after a unimodal rainy season [11]. As a result, local communities are enforced to depend on trees and shrubs to feed their livestock in the coming dry seasons [11]. However, fodder trees and shrubs of the area along with the associated indigenous knowledge are shrinking due to different anthropogenic factors [12], which is similar to other semiarid rangelands of the country [8, 13, 14]. So, an ethnobotanical study is found to be crucial in order to conserve fodder plants and the associated indigenous knowledge from their total losses. In addition, interests in indigenous knowledge of fodder plants and their management in pastoral systems are increasing [15, 16]. Therefore, this study was designed with the objective of documenting fodder trees and shrubs and the associated indigenous knowledge in Metema District.

## 2. Methods

### 2.1. Description of the Study Area

The study was conducted in Metema District located in northwestern Ethiopia at a far‐flung distance of about 900 km from the country′s capital city, Addis Ababa at 12°40 ^′^00 ^″^ N and 36°8 ^′^00 ^″^ E. It belongs to the West Gondar Zone centered Genda Wuha Town. The district harbors a total area of about 440,000 ha partitioned into two towns and 17 rural kebeles with more than 60 km international boundary with the Republic of Sudan [11].

The district is largely a lowland rangeland with spaced plateaus with an altitudinal range of 550–1608 m above sea level with a hotter climate [17]. Analysis of the meteorological data showed that the mean annual temperature of the area is about 26.2°C, and its rainfall is unimodal restricted only for 3–4 months (June to September) with a mean of 1008 mm. The soil type is predominantly black with vertic properties. Natural vegetation of the district is mainly composed of *Combretum*–*Terminalia* broad‐leaved deciduous woodland type with different *Acacia* species [11, 18]. According to the last census of the country [19], total inhabitants of the district were 110,231 with an increase of 100.78% over the 1994 census. They were from different ethnic groups including Amhara, Gumuz, Agaw, and Kemant [11] and following Ethiopian Orthodox Christianity as a dominant (83.4%) religion [18, 19].

### 2.2. Study Site and Informant Sampling Techniques

**Figure 1:**
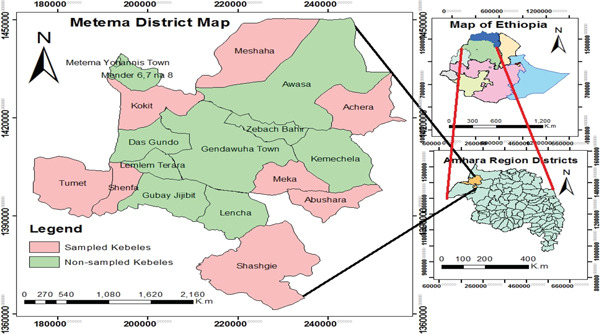
Location map of Metema District.

General information about the study area was obtained from Metema District Administration officials, agricultural officials, and recommended knowledgeable inhabitants. Then, eight sample kebeles (Figure 1) were selected purposively based on better livestock production and vegetation availability following Martin [20]. The sample size of informants was determined by using Cochran′s formula [21].

n=Z2.P.1−Pe2,

where *n* is the required sample size, *Z* is the *Z*‐value at 95% confidence level, *P* is the estimated proportion of the population (0.5), and *e* is the desired margin of error (5*%* = 0.05).

Accordingly, a total of 385 informants, composed of 40 knowledgeable key informants and 345 general informants, were selected.

n=1.962∗0.5∗10.5−0.052≈385.

Purposive sampling method was used to select key informants. To select the general informants, 345 households were selected through the systematic random sampling method. Then after, one individual per household who had close interaction with livestock and was aged above 20 years was taken as a general informant following Kiptot [16]. The lower age boundary of 20 years is based on the recommendations of Balick and Cox [22] in order to get enough and accurate data since they are expected to acquire enough ethnobotanical knowledge of the area.

### 2.3. Ethnobotanical Data Collection Methods

Data collection was carried out in two rounds: one was during the driest and fodder‐scarce time (March to May 2021) and the other was during the presence of ample fodder (August to December, 2022). Individual interviews, guided field walks, and focus group discussions were applicable data collection methods through checklists of semistructured questionnaires [20, 23]. Individual interviews were performed with both general and key informants focused on informants′ demographic profiles, including livestock composition both in type and quantity. The interviews also emphasized fodder trees and shrubs, local names, parts used, types of consuming animals, and conditions of utilization. Guided field walks were considered to collect data on plant habit, habitat, specimen collection, environmental threats, and additional information that was not addressed during individual interviews. Specimen identification was made at the University of Gondar, Ethiopia, by using published volumes of the Flora of Ethiopia and Eritrea. To check the validity of information and to seek further information, focus group discussions with selected key informants were conducted. All the methods mentioned above were performed following prestarting authors [20, 22, 23].

### 2.4. Ethnobotanical Data Analysis

Descriptive statistics were used to organize and analyze collected data. Indigenous knowledge distribution between age groups and informant types (key and general informants) was analyzed using SPSS Version 26.0 software. Jaccard′s coefficient of similarity (JCS) of fodder species composition between the present study area and other previous studies having similar agroecologies was compared following Kent and Coker [24]. JCS was calculated for paired habitat types (A and B) as follows:

JCS=cc+b+a,

where *a* is the number of species found only in Habitat A, *b* is the number of species only in Habitat B, and *c* is the number of common species in Habitats A and B.

Validity and knowledge homogeneity within the community were also analyzed using different ethnobotanical data ranking and scoring methods (preference ranking, paired comparison, and relative frequency of citation [RFC]) [20, 23]. Key informants were allowed to select six of the most preferred fodder species by livestock, and then preference ranking was made on the selected plants based on their palatability using eight well‐experienced key informants. The highest value (6) was given to the most preferred species and the lower value (1) to the least preferred species. Likewise, key informants were invited to select five fodder species based on milk and meat production, and then the species were ranked by seven key informants using pair‐wise comparison in order to test the consistency of the relationships. The possible number of pairs was calculated using the following formula:

Number of pairs=nn−12,

where *n* is number of plant species compared.

RFC was used to rank the citation frequency of fodder plants to analyze their relative importance following Tardio and Pardo‐de‐Santayana [25]. Specifically, a higher RFC value indicates a higher frequency of utilization for the plant. RFC was calculated as

RFC=FCN01<FC<,

where FC is the number of informants that mentioned the use of the species as fodder and *N* is the total number of informants included in the study. We used RFC values to rank the most cited fodder plants.

## 3. Results and Discussion

### 3.1. Sociodemographic Profile of Informants

A total of 385 informants (345 general and 40 key informants) were sampled from the study area (Table 1). Informants were aged from 20 to 81 years, of which 197 (51.2%) were included under the age range of 51–81 years and the rest 188 (48.8%) were included under the age range of 20–50 years. Related to sex, all the selected informants were males except for 11 general informants who were females (Table 1). This is because in Metema District communities, the responsibilities of livestock management are held mostly by men, and thus ethnobotanical fodder knowledge is expected to be concentrated in males rather than females. In addition, females were involuntarily interviewed due to cultural influence. This is in line with the Maasai pastoralists of Kenya [16].

**Table 1:** Age and sex structure of general and key informants interviewed.

**Informant type**	**Sex**	**Age group in years**		**Total**
		**20–50**	**51–81**	
Key informant	Male	18	22	40
	Female	—	—	—
	Total	18	22	40

General informant	Male	163	171	334
	Female	7	4	11
	Total	170	175	345

Grand total		188	197	385

### 3.2. Livestock Production and Management Systems

Livestock production is the main source of livelihood for Metema District communities. Livestock reared in the district include cattle, goats, sheep, and donkeys, and each household was reported to have livestock (Table 2) which is in agreement with other reports [8, 16]. The average number of goats per household was 34, cattle were 26, sheep were four, and donkeys were two. The maximum number of livestock stands per household was recorded for goats and cattle, which were 249 and 124, respectively. Camel was the least livestock population in the area and also not herded in all households. As a result, its average number per household was below one (0.25). Cattle are kept for cash income, milk, meat, and land plowing. Goats and sheep were herded for meat and cash income values, while donkeys and camels were used for transportation and carrying loads.

**Table 2:** Livestock production and management systems in Metema District.

**Livestock type**	**Livestock number per household in range**	**Average livestock per household**	**Livelihood role**
Cattle	1–124	26	Cash income, milk, meat, and plowing
Goat	3–249	34	Meat and cash income
Sheep	0–83	4	Meat and cash income
Donkey	0–5	2	Transportation and carrying loads
Camel	0–3	0.25	Transportation and carrying loads

Accessibility of fodder and forage in the district was dependent on the season. Data from the informants and field observations showed that plenty of fodder and forage was available 3 weeks after the first shower of rain (June or July depending on the rain) to the mid of February. But in the rest of the three dry months (March to May), livestock could not get enough forage due to resource scarcity. Some farmers had experience collecting a limited amount of forage grasses in their homesteads during the accessible period. During the dry periods, some lactating cows and selected oxen were allowed to feed on the forage collected at home. In contrast, all other livestock had to forage for trees and shrubs away from the house.

### 3.3. Fodder Trees and Shrubs

In the district, a total of 46 fodder tree and shrub species included in 36 genera and 24 families were identified (Table 3). These consisted of 36 trees and 10 shrubs. Even though the number of species recorded is remarkable, it was found to be somewhat lower than the browse species recorded in semiarid areas of Awash National Park, which were 69 trees and shrubs [5]. This difference in species number might be due to the presence of better conservation efforts in the park area than in the current study area. Likewise, more species (47 browsed species) were recorded in the southern Zone of Tigray characterized by having different agroecologies [26]. Conversely, fewer fodder trees and shrubs were recorded in the semiarid regions of northern Ethiopia [27], Ethiopia′s mid‐rift valley [9], and the Wolayta zone of Ethiopia [28] which had 20, 18, and 28 species, respectively. This indicated that Metema District harbors a good number of fodder trees and shrubs in comparison with some other areas with similar agroecologies.

**Table 3:** List of fodder trees and shrubs in Metema District.

**Scientific name**	**Family**	**H**	**Ht**	**PP**	**No. R.**	**RFC**	**CA**
*Acanthus sennii* Chiov.	Acanthaceae	S	W	L	71	0.18	G
*Adansonia digitata* (L.) Del.	Moraceae	T	W	L, F, and tw	102	0.26	G and Ca
*Anogeissus leiocarpa* (A. Rich) Guill. & Perr	Combretaceae	T	W	L and tw	296	0.77	Ct, G, Ca, and Sh
*Azadirachta indica* A. Juss.	Meliaceae	T	H	L	25	0.06	G and Ca
*Balanites aegyptiaca* L.	Balanitaceae	T	W	L and tw	209	0.54	Ct, G, Ca, and Sh
*Boswellia papyrifera* Hochst. ex A. Rich	Burseraceae	T	W	L	61	0.16	G
*Carissa spinarum* L.	Apocynaceae	S	W	L	57	0.19	G
*Combretum hartmannianum* Schweinf.	Combretaceae	T	W	L	18	0.05	G and Ca
*Combretum molle* R.Br. ex G. Don	Combretaceae	T	W	L	136	0.35	G and Ca
*Combretum* sp Fresen	Combretaceae	T	W	L	97	0.25	G and Ca
*Cordia africana* Lam.	Boraginaceae	T	H	L and F	111	0.29	Ct, G, and Ca
*Crateva adansonii* DC.	Rubiaceae	T	W	L	106	0.28	G
*Dalbergia melanoxylon* Guill. & Perr.	Fabaceae	T	W	L	53	0.18	G and Ca
*Dichrostachys cinerea* Wight & Am	Fabaceae	S	W	L	297	0.77	Ct, G, and Ca
*Diospyros abyssinica* (Hiem) F.	Ebenaceae	T	W	L and F	61	0.19	G and Ca
*Diospyros mespiliformis* (Hiem) F.	Ebenaceae	T	W	L and F	99	0.26	G and Ca
*Dombeya torrida* (J.F. Gmel.) P. Bamps	Sterculiaceae	T	W	L	66	0.17	G and Ca
*Ekebergia capensis* Sparrm	Meliaceae	T	W	L	86	0.22	G and Ca
*Ficus sycomorus* L.	Moraceae	T	W	L and F	94	0.24	Ct, G, Ca, and Sh
*Ficus sur* Forssk.	Moraceae	T	W	L and F	153	0.40	Ct, G, Ca, and Sh
*Ficus thonningii* Blume	Moraceae	T	W	L, tw, and B	251	0.65	Ct, G, Ca, and Sh
*Ficus vallis-choudae* Del.	Moraceae	T	W	L and F	174	0.45	G, Ca, and Sh
*Flueggea virosa* Yebaria Guill. & Perr	Euphorbiaceae	T	W	L	198	0.51	Ct, G, and Ca
*Grewia ferruginea* Hochst. ex A. Rich	Tiliaceae	S	W	L and tw	162	0.42	Ct, G, Ca, and Sh
*Lannea fruticosa* (Hochst. ex a. Rich) Engle	Anacardiaceae	T	W	L and F	146	0.38	Ct, G, Ca, and Sh
*Melia azedarach* L.	Meliaceae	T	H	L	48	0.12	G and Ca
*Moringa stenopetala* L.	Moringaceae	T	H	L and F	29	0.08	G
*Maytenus undata* (Thunb.) Blakelock	Celastraceae	S	W	L	181	0.47	Ct and G
*Oxytenanthera abyssinica* (A. Rich) Mumro.	Poaceae	S	W	L	174	0.45	Ct, G, and Ca
*Polygala persicariifolia* DC.	Polygalaceae	T	W	L	59	0.15	G and Ca
*Pterocarpus lucens* Guill. & Perr.	Fabaceae	T	W	L and tw	367	0.95	Ct, G, and Ca
*Rhus glutinosa* Hochst. ex A. Rich	Anacardiaceae	S	W	L and tw	92	0.29	G and Ca
*Senegalia polyacantha* (Willd.) Seigler & Ebinger	Fabaceae	T	W	L, F, and tw	291	0.76	Ct, G, and Ca
*Senegalia senegal* (L.) Britton, Sci. Surv.	Fabaceae	T	W	L	284	0.73	Ct, G, and Ca
*Sida cuneifolia* Roxb.	Asteraceae	S	W	All	28	0.07	Ct, G, and Sh
*Sterculia setigera* Del.	Sterculiaceae	T	W	L	203	0.53	G and Ca
*Stereospermum kunthianum* Cham.	Bignoniaceae	T	W	L	86	0.22	Ct, G, and Ca
*Syzygium guineense* (Willd.) DC.	Myrtaceae	T	W	L	69	0.18	G
*Tamarindus indica* L.	Fabaceae	T	W	L and tw	97	0.25	Ct, G, and Ca
*Terminalia brownie* Pers.	Combretaceae	T	W	L	207	0.54	G and Ca
*Terminalia laxiflora* Engl. & Diels	Combretaceae	T	W	L	79	0.21	G and Ca
*Vachellia seyal* (Del.) P.J.H. Hurter	Fabaceae	T	W	L, F, and tw	209	0.54	Ct, G, and Ca
*Vachellia sieberiana* (DC.) Kyal. & Boatwr	Fabaceae	T	W	L and tw	226	0.59	G and Ca
*Ximenia americana* L.	Olacaceae	S	W	L	91	0.24	Ct, G, and Ca
*Ziziphus abyssinica* Hochst. ex A. Rich	Rhamnaceae	T	W	L	124	0.32	G and Ca
*Ziziphus spina-christi* (L.) Desf.	Rhamnaceae	S	W	L and F	331	0.86	Ct, G, and Ca

Abbreviations: B, stem bark; Ca, camel; CA, consuming animals; Ct, cattle; F, fruit/pod; G, goat; Hb, habit; H, homestead; Ht, habitat; L, leaves; No. R, number of reports; PP, useful plant part; S, shrub; Sh, sheep; T, tree; tw, twig; W, wild.

Fabaceae was found to be the most fodder species‐rich family (eight species, 17.39%), followed by Combretaceae (six species, 13.04%), Moraceae (five species, 10.87%), and Meliaceae (three species, 6.52%). Anacardiaceae, Ebenaceae, Sterculiaceae, and Rhamnaceae contained two species each (4.35% each). These eight families accounted for 65.22% of the total recorded fodder species. The remaining 16 families were represented by a single species each. Likewise, other studies elsewhere in Ethiopia also reported Fabaceae as the most fodder species‐rich family [8, 27]. The dominant fodder role of Fabaceae could be associated with its highest species diversity in Ethiopian Flora in addition to its wider global distribution.

### 3.4. JCS Result

JCS analysis showed that fodder species of Metema District had relatively the greatest similarity (18%) with fodder species of Awash National Park surrounding areas [8], followed by fodder species of Abergele District [27] (Table 4). This proved that there are certain homologies in the utilization of fodder species between the three areas. This similitude might be observed because of the agroecological similarity of the semiarid environments, whereas fodder plant species in the current study had the least similarity (5%) with fodder plants in Ethiopia′s mid rift valley [9]. This might be related to the difference in the agroecologies that harbor different fodder plant species in the two areas.

**Table 4:** JCS of fodder species between the current study area and other previous studies.

**Study area**	**Awash National Park**	**Mid rift valley**	**Southern Tigray**	**Wolayta zone**	**Abergele District**
Metema District	0.18 (18%)	0.05 (5%)	0.07 (7%)	0.07 (7%)	0.14 (14%)

### 3.5. Indigenous Knowledge of Fodder Trees and Shrubs

**Figure 2:**
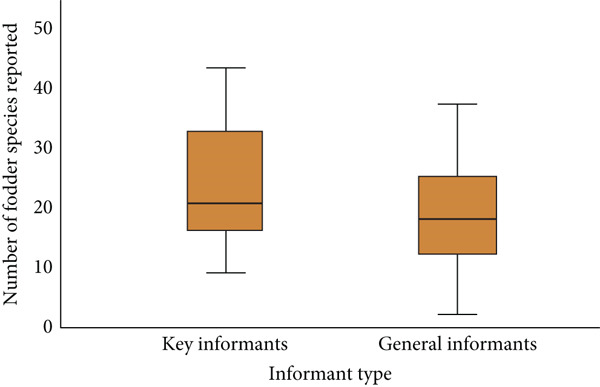
Simple boxplot of number of fodder plant species reported by informant types.

In Metema District, there was a significant indigenous knowledge variation between general informants and key informants (Table 5). Key informants were more knowledgeable than general informants, which is in line with the expected ethnobotanical knowledge [20]. The minimum and maximum number of fodder species reported by key informants was nine and 43, respectively, while it was two and 37 in the case of general informants (Figure 2). As they reported, key informants had life long experience and spent most of their time through livestock herding compared to general informants, and this might be the cause of the knowledge variation.

**Table 5:** Independent sample *t*‐test of significance on fodder plants by informant groups.

**Parameter**	**Informant group**	**N**	**Std. deviation**	**Mean**	**t** ** value** ^∗∗^	**p** ** value**
No. Sp. reported	Key informants	40	10.354	23.63	3.986	0.001
	General informants	345	7.669	18.31		

*Note:* Significant difference (*p* < 0.05)

Abbreviations: *N*, total number of informants; No. Sp. reported, number of fodder species reported.

^∗∗^ *t* (0.05) two‐tailed.

**Figure 3:**
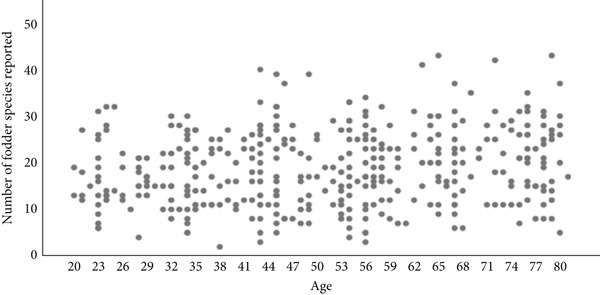
Scattered plot showing Pearson′s correlation between informants′ age and number of fodder plants listed.

The free plant list length also indicated a weak positive Pearson′s correlation between fodder plants knowledge and age of respondents (*r* = 0.19, *p* < 0.05). The free plant list length ranges from two to 43 fodder trees and shrubs (Figure 3). This might be linked with regular and direct contact of elders with fodder plants in their long‐lasting pastoral way of life, which provided them the opportunity to accumulate more knowledge than the younger individuals.

Related to the way of accessing plants for the livestock, key informants reported five types of accessing methods, while general informants reported only four accessing methods (Table 6). Four accessing methods (direct browsing, branch lopping, inclination, and tree shaking) were reported by more than or equal to 89.9% of key informants. But the maximum knowledge agreement on the fodder accessing method for livestock between general informants was 87.2%. This showed the presence of higher indigenous knowledge consistency between key informants than general informants. In contrast, cutting at the base of the trunk got the least agreement between key informants (5%) and higher agreement between general informants (52.6%). This indicated that key informants did not feed their livestock by cutting trees and shrubs at the trunk base. This might be done purposively to conserve the fodder plants as reported by a former author [8].

**Table 6:** Ways of accessing fodder plants to livestock by key and general informants.

**Ways of accessing the fodder**	**Number of reports per informant type (%)**	
	**Key informants**	**General informants**
Direct browsing	40 (100%)	301 (87.2%)
Lopping the branch	40 (100%)	237 (68.7%)
Cutting at the base of the trunk	2 (5%)	182 (52.6%)
Inclination	37 (92.5%)	54 (15.7%)
Shaking the plant	31 (89.9%)	0 (0%)

### 3.6. Sources of Fodders, Browsed Plant Parts, and Conditions Used

Most of the recorded fodder trees and shrubs (91.3%) were found in the wild area. The remaining four species (*Azadirachta indica*, *Cordia africana*, *Melia azedarach*, and *Moringa stenopetala*) which accounted for only 8.7% were available in homesteads (Table 3). These wild plant resources are communal properties of the local community for their fodder value especially during Ethiopian winter when no land area is covered by crops.

Leaves, fruits (pods), and twigs are plant parts used for fodder, which is in line with other study results [8, 9, 28]. Leaves of all recorded plants, twigs of 10 species, and pods of 9 species were browsed plant parts used as fodder by most livestock populations (Table 3). The dominance of leaves is in parallel to other studies elsewhere in Ethiopia [28–30].

Fresh and dried forms of leaves and pods were used by animals. In dried form, they became fodder sources after shedding during dry seasons when animals did not access the fresh form. Dry forms are highly preferred fodders especially for goats during dry times, but twigs are used only in fresh forms. This is supported by the report of Ebro [31] in the mid rift valley production system in which pods of *Acacia tortilis* were commonly consumed by animals.

### 3.7. Ethnobotanical Ranking and Scoring

**Figure 4:**
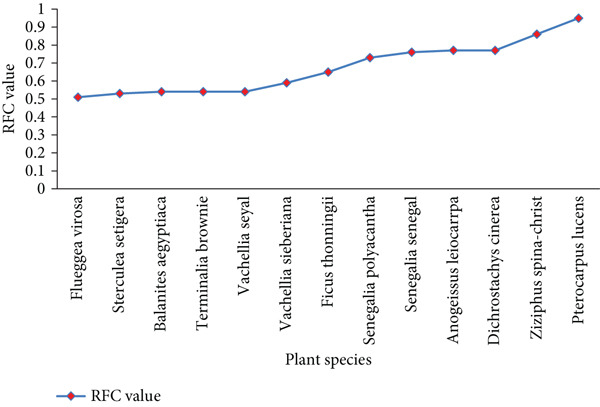
Fodder species with RFC value ≥ 0.5 in the study area.

RFC of fodder species in the study area ranged from 0.05 to 0.95 (Table 3) with an average of 0.36. Thirteen species had ≥ 0.5 RFC values, of which *Pterocarpus lucens* is the most consistent fodder species with an RFC value of 0.95 (95%), followed by *Ziziphus spina-christi* with an RFC value of 0.86 (86%) (Figure 4). The RFC values of these species indicated their preferential ability to fulfill fodder gaps in the area. Conversely, *Combretum hartmannianum* was the least reported plant species with an RFC value of 0.05 (5%), followed by *Azadirachta indica* with an RFC value of 0.06 (6%) (Table 3). This higher RFC value is an indicator of indigenous knowledge homogeneity among the livestock herders on selected fodder plant species. Less preference for *Azadirachta indica* for fodder might be related to its exotic nature. Exotic species have an experience of lowering herbivory in their new areas by escaping out from their co‐evolved natural enemies [32, 33].

*Pterocarpus lucens*, *Senegalia senegal*, and *Balanites aegyptiaca* were ranked one to three in respective order in pair‐wise comparison for milk and meat production (Table 7). In relation to their palatability, *Pterocarpus lucens* ranked first, while *Senegalia senegal* and *Balanites aegyptiaca* stood third and fourth following *Dichrostachys cinerea* (Table 8). The overall scoring and ranking analyses showed that *Pterocarpus lucens* was the most important fodder species with great consistency. Itis a deciduous shrub or tree species found in tropical semiarid environments [34]. Couteron et al. [35] also reported this plant as the most preferred fodder species by animals in Burkina Faso. This might be related to leaf nutrient content and the palatability nature of the plant. According to Ngom et al.′s [36] report, *Pterocarpus lucens* had a forage production ability of between 144.3 and 211.4 kg DM/ha with an average of 178 kg DM/ha in Northern Senegal. It was also reported as the most marketable fodder species in the Niono market of Mali, which accounts for 67.9% of the total marketable fodders [37]. Based on the authors′ estimation, about 458 tons of *Pterocarpus lucens* leaves are sold per year with a total cost of $10,734, which is 37.6% of the total fodder cash income.

**Table 7:** Paired comparison of five selected fodder plants based on milk and meat production.

**Scientific name**	**Key informants (I1–I7)**							**Total**	**Rank**
	**I1**	**I2**	**I3**	**I4**	**I5**	**I6**	**I7**		
*Pterocarpus lucens*	4	4	4	4	4	4	4	28	1^st^
*Senegalia senegal*	3	3	0	3	3	2	3	17	2^nd^
*Balanites aegyptiaca*	2	0	3	1	2	1	2	11	3^rd^
*Ziziphus spina-christi*	1	1	2	2	0	3	1	10	4^th^
*Flueggea virosa*	0	2	1	0	1	0	0	4	5^th^
Total	10	10	10	10	10	10	10	70	

**Table 8:** Preference ranking of six most frequently listed trees and shrubs for fodder based on preference by animals (6 = *most preferred*, 1 = *least preferred*).

**Plant species**	**Key informants (I1–I8)**								**Total**	**Rank**
	**I1**	**I2**	**I3**	**I4**	**I5**	**I6**	**I7**	**I8**		
*Pterocarpus lucens*	6	5	6	5	6	5	6	6	45	1^st^
*Dichrostachys cinerea*	5	6	5	4	3	6	5	3	37	2^nd^
*Senegalia senegal*	2	4	4	6	2	4	4	2	28	3^rd^
*Balanites aegyptiaca*	4	2	1	3	4	2	2	5	23	4^th^
*Anogeissus leiocarpa*	3	1	3	1	5	1	3	4	21	5^th^
*Ficus thonningii*	1	3	2	2	1	3	1	1	14	6^th^

Based on field observation and informants report, leaves of *Pterocarpus lucens* is used by livestock both in dried form after shedding and in fresh condition. Livestock′s feed shortage is a stress in the study area for at least 2 weeks duration starting from the first raining until grasses and other herbaceous species reached for grazing. During this critical time, *Pterocarpus lucens* develop new leaves 2–3 days after the first showering of rain and save the animals life mainly cattle from death.

### 3.8. Animals That Feed on Trees and Shrubs

**Figure 5:**
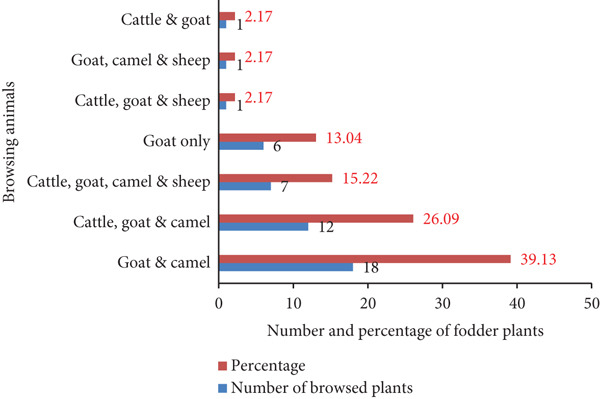
Number and percentage of fodder plants with browsing livestock types.

Camels, cattle, goats, and sheep are domestic animals in the area that feed on trees and shrubs. Jamala et al. [38] also reported that fodder trees and shrubs were noted to support livestock such as cattle, sheep, goats, donkeys, and camels in arid and semiarid environments. About 39.1% of fodder species were shared browses only by goats and camels, and 26.1% were shared only by cattle, goats, and camels, while 13.04% of plants are browsed only by goats (Figure 5). Bahru et al. [8] revealed similar results that most fodder species were consumed by goats and camels followed by goats, cattle, and camels in and around semiarid areas of Awash National Park. All the recorded fodder species (100%) were browsed by goats and camels, which browsed about 86.9% of them, whereas sheep browsed only 19.6% of fodder species (Table 3). Kiptot [16] also identified that all recorded fodder species in Kenya by Maasai pastoralists were browsed by goats. These might be related to the total dependency of goats and camels on browse plants year‐round, as Shenkute et al. [9] reported.

## 4. Conclusions

This study revealed that indigenous communities of Metema District possess high knowledge of tree and shrub fodder plants, which are vital components of their livestock feeding systems. The communities identified 46 fodder trees and shrubs, recognizing their source, valuable plant part, condition of consumption, and specific suitability for different livestock groups. The knowledge consistency of the reported fodder species between informants is relatively low with an average RFC of 0.36, which is below half (0.5). Significant differences in knowledge of fodder trees and shrubs were observed between key and general informants as well as age groups. The predominant methods of accessing fodder trees and shrubs included direct browsing, branch lopping, inclination, and shaking to mitigate fodder shortages. This study also identified some highly preferred trees and shrubs by the livestock including *Pterocarpus lucens*, *Senegalia senegal*, *Balanites aegyptiaca*, *Dichrostachys cinerea*, *Anogeissus leiocarpa*, and *Ficus thonningii*. These findings demonstrate that by exploring the rich but less studied treasure of fodder plants and their traditional knowledge, we can provide essential insights and information that may support sustainable livestock production.

## Ethics Statement

Since the knowledge of fodder plants was opened to any community with no secrets, neither ethical approval nor consent to participate was needed.

## Consent

The authors have nothing to report.

## Disclosure

The final manuscript was approved by all authors.

## Conflicts of Interest

The authors declare no conflicts of interest.

## Author Contributions

All authors had equal contributions in all of the tasks performed.

## Funding

The authors received no funding for this study.

## Data Availability

The data collected for this study were analyzed, interpreted, and incorporated in this manuscript.
